# Does the additional use of clomiphene citrate or letrozole for in vitro fertilization deserve more attention?

**DOI:** 10.1186/s12884-021-03668-x

**Published:** 2021-04-01

**Authors:** Ying Liang, Qing Guo, Xiao-Hua Wu, Li-Nan Zhang, Jun Ge, Mei-Ling Xu, Zheng-Li Feng, Xiao-Qian Wu

**Affiliations:** 1 Department of Reproduction Medicine, The Fourth Hospital of Shijiazhuang, Hebei Medicine University, Hebei, Hebei Province China; 2 Department of Pathology and Pathophysiology, Hebei Medicine University, Shijiazhuang, Hebei, China

**Keywords:** Letrozole, Clomiphene Citrate, ovulation induction, infertility, pregnancy

## Abstract

**Background:**

Adding clomiphene citrate (CC) and/or letrozole (LE) to in vitro fertilization (IVF) cycles for mild ovarian stimulation is a general approach. Although lots of researches have demonstrated partial benefits of the strategy, all-around effects of oral medications remained deficient. This paper aims to assess whether an addition of oral medication will result in considerable outcomes on T-Gn (total dose of gonadotropin), Gn days, total retrieved ova, high quality embryos, blastocyst number, ovarian hyperstimulation syndrome (OHSS) rate, clinical pregnancy rate and cumulative pregnancy rate, even if it was not conventional mild/minimal stimulations.

**Results:**

Participants were categorized to three diverse populations as high responders, normal responders and poor responders according to basal antral follicle count. T-Gn in patients treated with CC/LE distinctly decreased from 2496.96 IU/d to 1827.68 IU/d, from 2860.28 IU/d to 2119.99 IU/d, and from 3182.15 IU/d to 1802.84 IU/d, respectively. For high ovary responders and normal responders, the OHSS incidence rate also declined from 29.2 to 4.3% (*P* < 0.001) and from 1.1 to 0.0% (*P* = 0.090). Other, there was no statistical difference with respect to the T-retrieved ova (total retrieved ova), high quality embryos, cultured blastocyst and blastocyst number in high responders. For normal responders and poor ovary responders, T-Gn, Gn days, T-retrieved ova, high quality embryos, cultured blastocyst and blastocysts number in oral medications group all apparently decreased. Clinical pregnancy rate per fresh cycle of poor responders with prior oral medications was significantly decreased (25.7% vs. 50.8%, *P* = 0.005), and no significant differences in high responders and normal responders were expressed (52.5% vs. 44.2%, *P* = 0.310; 51.9% vs. 42.4%, *P* = 0.163) between two groups of participants. The numbers of cumulative pregnancy rates were lower in the conventional group compared to the add group for high (75.90% versus 81.03%, *P* = 0.279), normal (62.69% versus 71.36%, *P* = 0.016) and poor (39.74% versus 68.21%, *P* < 0.001) responders.

**Conclusions:**

The addition of CC/LE to the ovulation induction during IVF has certain efficacy in terms of low cost, low OHSS incidence. CC/LE deserves more recommendations as a responsible strategy in high responders due to advantageous pregnancy outcomes. For normal responders, the strategy needs to be considered with more comprehensive factors.

## Introduction

Controlled ovarian stimulation (COS), one of the key processes of assisted reproductive technology, increases the number of oocytes and embryos in the cycle and the pregnancy rate. Classic gonadotropin (Gn) injections brought great economic and psychological pressure to patients [1, 2], so the combined oral medicine program sprung up. Clomiphene citrate, competitively binds to estrogen receptors, to inhibit the negative feedback of estrogen to the hypothalamus and pituitary, promoting the release of endogenic FSH to promote follicle development [3]. Letrozole blocks estrogen synthesis by inhibiting aromatase activity, lowering its level in the blood. At the same time, it can block the conversion of androgens to estrogen at ovarian levels, and result in a short build-up of androgens in the ovaries. The accumulated androgens can also improve the ovary’s hormone response through the IGF-1 system at the outer weekly level [4].

The oral medications were previously used to induct ovulation, and were gradually replaced by FSH in pursuit of follicle averaging until the idea of micro/mild stimulation was proposed. Evidently, mild strategy has been being paid much attention because of its economic friendliness and convenience [5]. Since then, a large number of explorations have been made. One group of experts tried to make it easier for clinicians on ISMAAR meeting, where several terminologies were adopted internationally in 2007 but could not take into account the individual differences caused by age, ovarian reservation, BMI, etc. [6]. Nargund, Zegers-Hochschild et al. [6, 7] intended to limit the number of retrieved ova to fewer than eight for mild ovarian simulation IVF, because the less retrieved ova means the lower the risk of happening OHSS. Many studies have limited the use of Gn to 150 IU/d for mild stimulation/ minimal stimulation [8–11]. Actually, mild stimulation still seems to be a vague concept without a strict criterion worldwide.

Oral medications experienced a long but significant process on ovulation induction [12, 13], from single medicine for five consecutive days to a combination with Gn/HMG. Many studies often revealed the dilemma of choosing the particular protocol or a single population most likely to result in a partial assessment, as oral medication cycle becomes increasingly more universal [14–20]. Our study retrospectively analyzed 2724 patients who were treated by IVF-ET in reproductive medicine center of Shijiazhuang fourth Hospital from January 2017 to December 2018. To obtain data for clinical practices, we classified them according to whether they were proposed CC/LE in the process of ovulation induction. We ultimately got the clinical and laboratory outcomes.

## Materials and methods

### Participants

Women in 23 ~ 42 years old who had given their consent to ovarian stimulation for IVF-ET or ICSI-ET at this center from January 2017 to December 2018 were concluded. A total of 2724 cycles containing 1409 fresh transplantation cycles were obtained. The infertility years were 1 ~ 13 years. The causes of infertility included pelvic fallopian tube factors, male infertility, endometriosis, ovulation disorders and unexplained infertility. Poor responders were defined as patients whose basal antral follicle count (AFC) were less than five referring to the Bologna Criteria [21]. We classified high responders as patients with 15 and more basal antral follicles, and normal responders as 5 ~ 14 basal antral follicles. The comparison of clinical induction protocols included GnRH-a super long protocol, GnRH-a long protocol, GnRH antagonist protocol, minimal stimulation protocol, natural cycle protocol and so on.

For all participants, outcomes including age, BMI, T-Gn, T-retrieved ova, high quality embryos, cultured blastocyst, blastocysts number, and primary clinical outcomes including clinical pregnancy rate and cumulative pregnancy rate between the two groups with or without oral medication were presented.

For high (*n* = 573), normal (*n* = 1215) and poor (*n* = 727) ovarian responders, the above laboratory and clinical indicators were presented respectively. Moreover, we also analyzed the differences between single CC (*n* = 604) and single LE (*n* = 112) additions in laboratory and clinical outcomes.

The occurrence rates in high and normal responders of moderate and severe ovarian hyperstimulation syndrome (OHSS) were detected according to a modern classification [22].

### Group

Conventional group: routine ovulation induction by Gn/HMG, no oral CC or LE. Add group: extra CC and/or LE combined with routine Gn/HMG. Each subgroup (high responder; poor responder; normal responder) was divided into Conventional group and Add group according to the same criteria.

### Treatment procedure

In conventional group, the dosages (150 ~ 300 IU/d) of Gn were carried by routine dose according to the patients’ age, primary disease, ovarian reserve, body mass index (BMI) and so on.

In add group, we started with CC (or LE), 50–100 mg/d × 5d (2.5 mg/d × 5d) since Day 2–5. The dosages (150 ~ 300 IU/d) of Gn were adjusted according to the patients’ different conditions (starting dose, starting time, injection QD/QOD, etc.).

### Embryos obtaining

When diameter ≥ 18 mm of 60% follicles, 5000 ~ 10,000 U HCG was injected to induce ovulation. After 36 ~ 38 h, the oocytes were taken under the guidance of trans-vaginal ultrasound. We used conventional IVF or ICSI for fertilization. According to embryo grading standard [23], grade 1 ~ 3 embryos were available embryos. The embryos on day 3 of grade 1, 2 containing 7 ~ 9 homogeneously sized cleavage spheres (and the fragments≤20%) were high quality embryos. In fresh cycles, 1 ~ 2 cleavages or blastocysts were transferred. Progesterone 60 mg/d was intramuscularly injected for luteal support. The rest were frozen at cleavage stage or kept on being cultured until blastocysts.

### Reproductive outcomes

Clinical pregnancy rate, one of the pregnancy outcomes, was defined according to the International Glossary on Infertility and Fertility Care as the presence of ultrasonographic visualization of one or more gestational sacs or definitive signs of pregnancy [24]. Cumulative pregnancy obtained with fresh or vitrified embryos from the same stimulation cycle was defined when the pregnancy had achieved the presence of ultrasonographic visualization of one or more gestational sacs or definitive signs of pregnancy [25].

### Statistical analysis

We used student test to analyze hormones, age, BMI, T-Gn, T-retrieved ova, high quality embryos, cultured blastocyst and blastocysts number, which were recorded as the mean ± SD in each group. The clinical pregnancy rate and cumulative pregnancy rate were tested by chi square test. Statistical processes were performed by SPSS21.0 (SPSS lnc. Chicago, IL, USA) software at a two-sided significant level of 0.05.

## Results

### Comprehensive analysis with or without oral medications

**Fig. 1:**
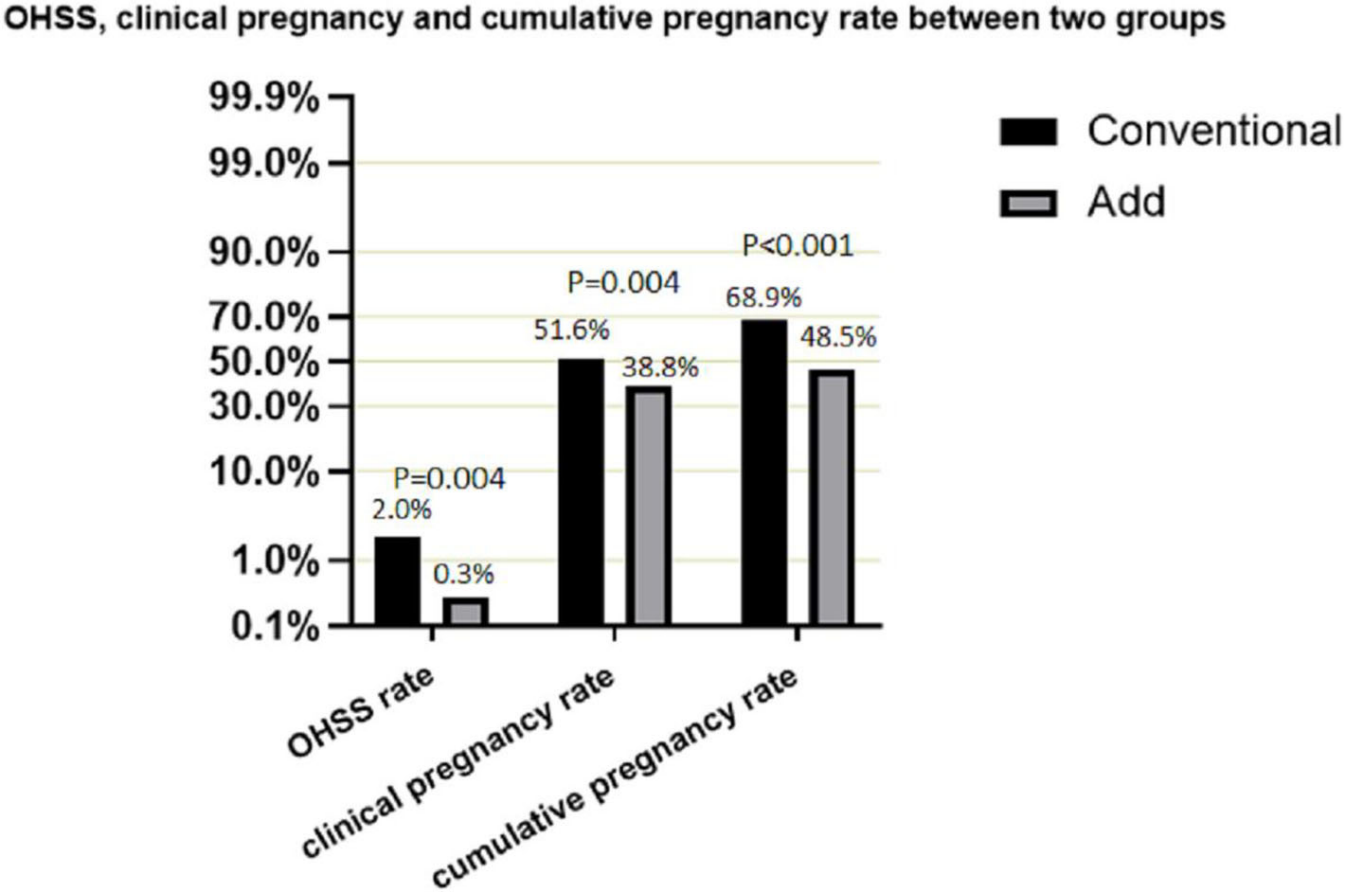
Chi square test showed statistical difference in OHSS rate, clinical pregnancy rate and cumulative pregnancy rate between Conventional group and Add group

As shown, there was no significant difference in the data of BMI, P, T-retrieved ova between the two groups (Table 1, Table 2). FSH, E2 and age in Add group were all significantly higher than that of Conventional group. Compared with Conventional group, many values in Add group were significantly adverse, such as LH, T, endometrial thickness, T-Gn, Gn days, T-retrieved ova, available embryos, high quality embryos, cultured blastocyst and blastocysts number (Table 1, Table 2). We found a decrease on OHSS rate, clinical pregnancy rate during the fresh cycle and cumulative pregnancy rate in the add group with oral administration (Fig. 1).

**Table 1:** Basal conditions between conventional group and add group

	N	Age	BMI	FSH	E2	P	LH	PRL	T
**Conventional**	**2096**	**30.15 ± 0.09**	**24.00 ± 0.09**	**5.39 ± 0.08**	**63.24 ± 5.09**	**1.00 ± 0.08**	**4.70 ± 0.11**	**16.80 ± 0.86**	**1.57 ± 0.08**
**Add**	**628**	**32.93 ± 0.19**	**23.66 ± 0.16**	**7.17 ± 0.18**	**114.07 ± 15.49**	**1.19 ± 0.18**	**4.16 ± 0.15**	**14.86 ± 0.40**	**1.17 ± 0.09**
***P*-value**	**–**	< 0.001*	**0.081**	< 0.001*	< 0.001*	**0.349**	0.011*	0.041*	0.009*

**Table 2:** Clinical and laboratory outcomes between conventional group and add group

	T-Gn	Gn days	T-retrieved ova	Available embryos	High quality embryos	Cultured blastocyst	Blastocyst number
**Conventional**	**2859.32 ± 26.20**	**12.40 ± 2.94**	**10.45 ± 0.17**	**5.45 ± 0.08**	**3.83 ± 0.07**	**6.00 ± 0.12**	**2.78 ± 0.07**
**Add**	**1858.52 ± 38.95**	**8.78 ± 3.24**	**9.74 ± 0.37**	**2.90 ± 0.11**	**2.01 ± 0.10**	**2.73 ± 0.17**	**1.39 ± 0.10**
***P*-value**	< 0.001*	< 0.001*	**0.077**	< 0.001*	< 0.001*	< 0.001*	< 0.001*

* Statistically significant. LH, FSH, E2, P, PRL, T: basal luteinizing hormone, follicle stimulating hormone, estrogen, progesterone, prolactin, testosterone. T-retrieved ova: Total number of retrieved ova

### Analyze patients with different ovarian functions

**Fig. 2:**
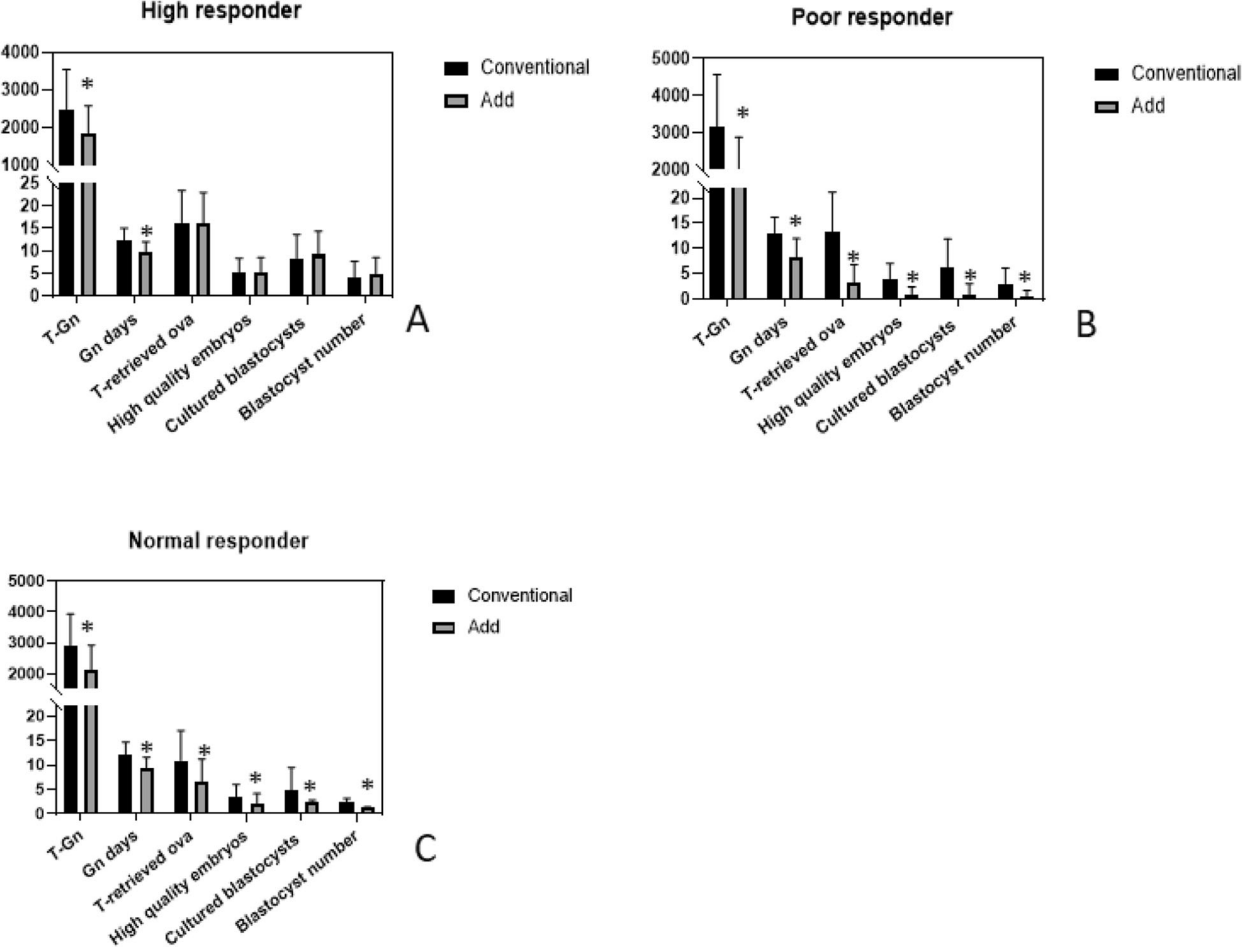
*P < 0.05. Conventional: no CC or LE. Add: CC and/or LE were added to induct ovulation

**Fig. 3:**
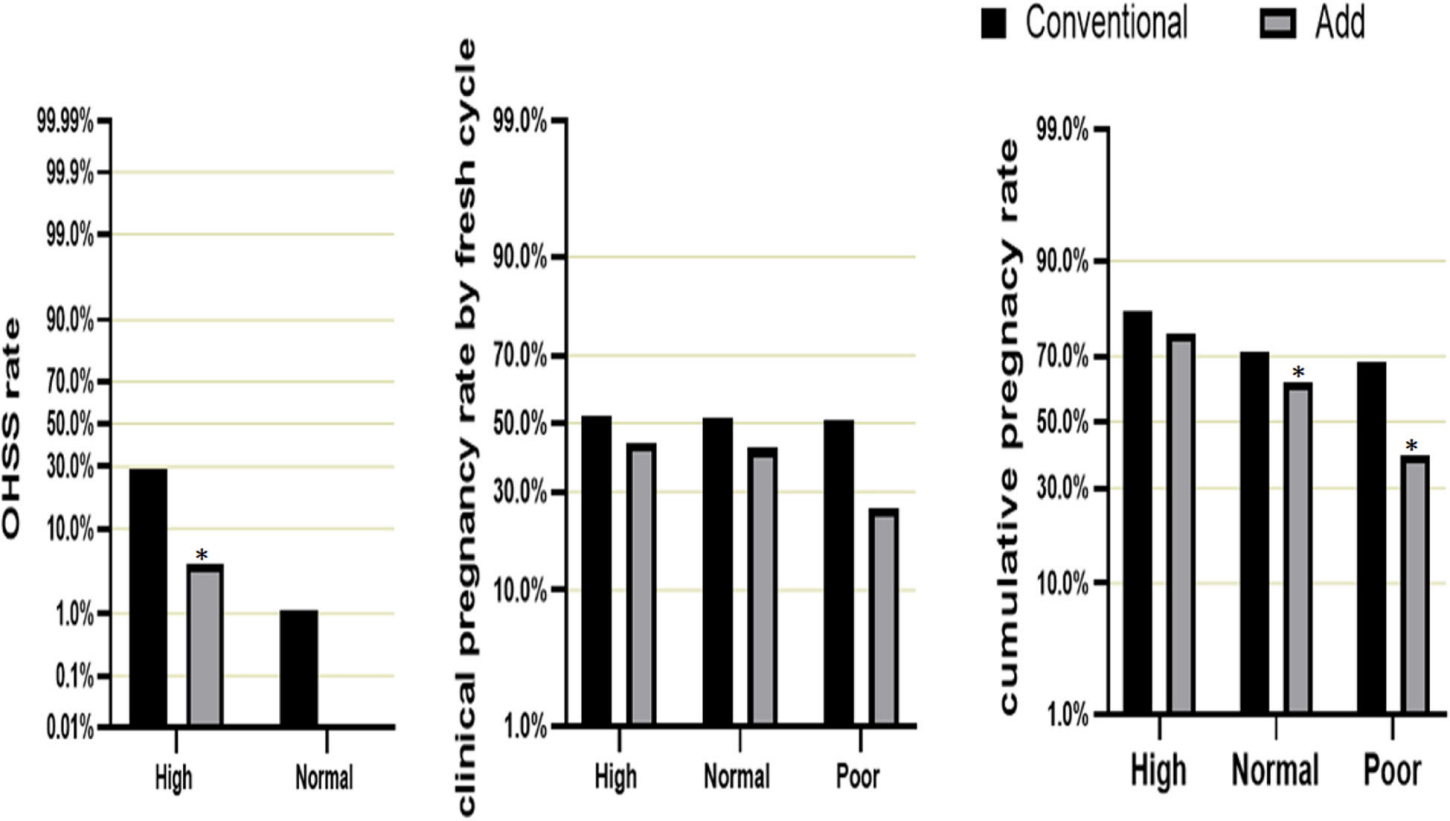
OHSS rates, clinical pregnancy rates by fresh cycle and cumulative pregnancy rates in high, normal and poor responders

In high ovary responders, T-Gn of those added oral medication distinctly decreased from 2496.96 IU/d to 1827.68 IU/d, *P* < 0.001. Other, there was no statistical difference in the T-retrieved ova, high quality embryos, cultured blastocyst and blastocyst number (Fig. 2a, Table 3). In poor ovary responders and normal responders, it should be noted that T-Gn, Gn days, T-retrieved ova, high quality embryos, cultured blastocyst, blastocysts number and OHSS rates in Add group all apparently decreased. Significantly, T-Gn respectively declined from 3182.15 IU/d to 1802.84 IU/d, 2860.28 IU/d to 2119.99 IU/d. (Fig. 2b, c;Tables 4, 5) Furthermore, for high ovary responders and normal responders, the OHSS incidence rate also declined from 29.2 to 4.3%, from 1.1 to 0.0% (Tables 4, 5; Fig. 3).

**Table 3:** Clinical and laboratory outcomes in high responders

	T-Gn	Gn days	Et	T-retrieved ova	High-quality embryos	cultured blastocyst	blastocysts number	OHSS rate	Pregnancy rate
**Conventional**	**2496.96 ± 1048.53**	**12.40 ± 2.94**	**11.23 ± 2.21**	**16.28 ± 7.27**	**5.08 ± 3.48**	**8.35 ± 5.35**	**4.09 ± 3.61**	**29.2%**	**52.5%**
**Add**	**1827.68 ± 761.26**	**9.71 ± 2.34**	**10.68 ± 1.99**	**16.13 ± 7.09**	**5.19 ± 3.46**	**9.19 ± 5.09**	**4.82 ± 3.91**	**4.3%**	**44.2%**
***P*-value**	< 0.001*	< 0.001*	**0.401**	**0.851**	**0.764**	**0.163**	**0.080**	< 0.001*	**0.310**

* Statistically significant. T-retrieved ova: Total number of retrieved ova. Pregnancy rate: Pregnancy rate per fresh cycle. Et:Endometrial thickness

**Table 4:** Clinical and laboratory outcomes in normal responders

	T-Gn	Gn days	Et	T-retrieved ova	High-quality embryos	cultured blastocyst	blastocysts number	OHSS rate	Pregnancy rate
**Conventional**	**2860.28 ± 1058.88**	**12.13 ± 2.53**	**11.81 ± 4.73**	**10.88 ± 6.14**	**3.38 ± 2.66**	**4.90 ± 4.58**	**2.22 ± 0.94**	**1.1%**	**51.9%**
**Add**	**2119.99 ± 792.95**	**9.40 ± 2.21**	**10.76 ± 2.39**	**6.54 ± 4.65**	**2.05 ± 2.11**	**2.50 ± 0.28**	**1.23 ± 0.12**	**0.0%**	**42.4%**
***P*-value**	< 0.001*	< 0.001*	**0.093**	< 0.001*	< 0.001*	< 0.001*	< 0.001*	**0.090**	**0.163**

* Statistically significant. T-retrieved ova: Total number of retrieved ova. Pregnancy rate: Pregnancy rate per fresh cycle. Et:Endometrial thickness

**Table 5:** Clinical and laboratory outcomes in poor responders

	T-Gn	Gn days	Et	T-retrieved ova	High-quality embryos	cultured blastocyst	blastocysts number	Pregnancy rate
**Conventional**	**3182.15 ± 1392.687**	**12.93 ± 3.23**	**11.67 ± 5.55**	**13.14 ± 8.05**	**3.70 ± 3.27**	**6.11 ± 5.75**	**2.72 ± 0.37**	**50.8%**
**Add**	**1802.84 ± 1081.352**	**8.22 ± 3.72**	**10.71 ± 2.23**	**3.19 ± 3.58**	**0.99 ± 0.12**	**0.85 ± 0.18**	**1.38 ± 0.11**	**25.7%**
***P*-value**	< 0.001*	< 0.001*	**0.309**	< 0.001*	< 0.001*	< 0.001*	< 0.001*	0.005*

* Statistically significant. T-retrieved ova: Total number of retrieved ova. Pregnancy rate: Clinical pregnancy rate per fresh cycle. Et:Endometrial thickness

Secondly, effects of CC or LE on the clinical pregnancy rate of patients with different ovarian function varied during fresh cycle transplantations. Results of chi square test showed that clinical pregnancy rate of poor responders with prior oral medications was significantly decreased (25.7% vs. 50.8%), and no significant differences in high responders and normal responders were expressed (52.5% vs. 44.2%; 51.9% vs. 42.4%) (Fig. 3).

The numbers of cumulative pregnancy rates were lower in the conventional group compared to the add group for high (75.90% versus 81.03%, *P* = 0.279), normal (62.69% versus 71.36%, *P* = 0.016) and poor (39.74% versus 68.21%, *P* < 0.001) responders (Fig. 3).

### Simple comparison about the difference between CC and LE

In CC group, compared with the LE group, the T-Gn decreased evidently, while the T-retrieved ova, high quality embryos, cultured blastocyst, blastocysts number and pregnancy rate increased. The differences were statistically significant. And OHSS rate between the two groups indicated no difference (Table 6). Adding CC alone was maybe prior to LE alone.

**Table 6:** Laboratory and clinical outcomes in CC or LE

	N	T-Gn	T-retrieved ova	High quality embryos	Cultured blastocyst	Blastocyst number	OHSS rate	Pregnancy rate
**CC**	**604**	**1787.15 ± 37.94**	**6.40 ± 0.27**	**2.05 ± 0.10**	**2.75 ± 0.17**	**1.40 ± 0.11**	**0.8%**	**44.1%**
**LE**	**112**	**2092.25 ± 111.22**	**2.93 ± 0.25**	**0.78 ± 0.13**	**0.69 ± 0.16**	**0.32 ± 0.10**	**1.1%**	**15.4%**
***P*-value**	**–**	< 0.001*	< 0.001*	< 0.001*	< 0.001*	< 0.001*	**0.339**	0.007*

* Statistically significant. T-retrieved ova: Total number of retrieved ova. Pregnancy rate: Pregnancy rate per fresh cycle

## Discussion

To reduce FSH dose, a series of trials compared CC/LE with Gn versus Gn, wondering if oral regimen was as effective as Gn alone. There was a common view having limited the use of Gn to 150 IU/d for mild stimulation and such a low dose did not stress side effects on pregnancy outcome in a number of randomized trials. One of them revealed that the ongoing pregnancy rate for mild ovarian stimulation (150 IU/d alone) was 12.8% versus 13.6% for conventional ovarian stimulation (450 IU/d) in poor responders (*P* > 0.05) [26]. In our statistical analysis, we analyzed the significance of adding oral medications, rather the fixed dose of exogenous Gn, and supplied the edges of adding oral agents to the ovulation process in different populations.

CC or LE regimens, which were associated with a reduction in the incidence of OHSS and low costs although by low-quality evidences, benefitted the poor or normal response populations [27–29]. In our research, for high ovary responders and normal responders, we can see that the OHSS incidence rates extremely declined. In patients expected to be normal responders, Siristatidis et al. proved laboratory outcomes including the total dose of Gn administered and retrieved ova were significantly lower than conventional group, which was consistent with our conclusion [30]. The adjunctive use of CC in IVF produced good efficacy for lowering the Gn level by a retrospective study covering 77 patients with POR [31]. No regardless of populations in our study, fewer Gn days thereby lower T-Gn and lower costs were required for ovarian stimulation in Add group patients compared with those in Conventional group. Although without a strict criterion (150 IU/d), the strategy for using CC/LE during IVF cycles was to develop such a patient-friendly stimulation that costs were reduced by decreasing the total dose of Gn compared with conventional ovarian stimulation. All in all, addition of CC or LE exactly led to a cut-down in the total Gn, total costs and the OHSS incidence rates.

According to high responders, one recent study had identified that the addition of LE was prior to without LE group depending on higher metaphase II and fertilized oocytes retrieved and similarly clinical pregnancy rates. However, a reduction of clinical pregnancy rates and live birth rates in letrozole group led the role of LE in ovarian stimulation of high responders to be controversial [32–34]. In our research, only 2 high response cases used LE alone as additional medication. With the addition of CC, our high response results came from most (91/93) cases along in our study, showing that adding CC to the ovulation process reduced Gn consumptions by 24.5%, without reducing other indicators including clinical pregnancy rate. Overall, CC regimen for high responders is recommended further exploration. It is obvious that current researches on the application of CC / LE in IVF cycle are mainly aimed at patients with normal response and low response, and the benefits of CC in high responders are lacking.

Although CC or LE alone has achieved good results in ovarian stimulation for women underwent an-ovulatory infertility, especially PCOS, CC resistance is inevitable. At present, the main solutions to the problem are exogenous Gn therapy and laparoscopic ovarian drilling [35, 36]. In order to save time and avoid more aggressive surgeries, some experts have come up with a combination of CC and LE in infertile patients with CC resistant polycystic ovary syndrome as a novel insight [37, 38]. Under the condition of very low ovarian response in our study, seven cases were treated with CC combined with LE, and one of the three individuals got a high-quality embryo after four times of ova retrieval and two times of transplantation, and finally successful conceived. Therefore, more attention can be paid to the joint CC plus LE strategy.

To estimate the clinical efficacy, related primary outcomes including clinical pregnancy rate and cumulative pregnancy rate were often referred [38, 39]. A cochrane systematic review reported eight RCT studies including clinical pregnancy rate, where no clear evidence of a difference was observed between protocols of CC or LE with or without Gn in conjunction with or without antagonist versus Gn (with GnRH agonist or antagonist) [28]. One RCT trial with 695 patients reported clinical pregnancy rates per transfer (23.2% vs 19.9%) and per cycle start (13.2% vs 15.3%) in poor responders [18]. Combining CC or LE with Gn was observed a lightly lower clinical pregnancy count for normal responders receiving mild versus conventional antagonist ovarian stimulation, which was consistent with our results [30]. Despite of the downward trend in terms of our clinical pregnancy rate, there was no significance between two groups for high responders under fresh cycles. Cumulative pregnancy rate, both the fresh and resuscitation cycles included, can accurately reflect the benefits of patients after one ova retrieval. The similar result of cumulative pregnancy rate in high responders (75.9% in add group versus 81.0% in conventional group) approved the conclusion that CC/LE was an effective means for clinical outcome, apart from the low cost and low OHSS rate. And the percentage of normal responders in add group was less than conventional group (62.7% versus 71.4%), suggesting that the diversity may be related to the less transfer number of available embryos. As for cumulative pregnancy rate of poor responders, similar to clinical pregnancy rate under fresh cycles, the percentage in add group was obviously lower than conventional group. We can’t ignore the fact that the poor responders who had obtained CC/LE during the medical treatment were likely the ones with worse response in the retrospective study, so the results should be considered more comprehensively and carefully. Further randomized clinical trials are needed to obtain a more effective recommendation.

## Data Availability

The datasets used and/or analyzed during the current study are available from the corresponding author on reasonable request.
